# Does immunohistochemical marker conversion affect the prognosis in breast cancer patients receiving neoadjuvant chemotherapy?

**DOI:** 10.1038/s41598-024-64492-9

**Published:** 2024-06-25

**Authors:** Mehmet Uzun, Elif Atag, Eda Caliskan Yildirim, Murat Keser, Huseyin Salih Semiz, Olcun Umit Unal

**Affiliations:** 1 Internal Medicine, Department of Medical Oncology, Dokuz Eylul University, Izmir, Turkey; 2 Department of Medical Oncology, Tepecik Training and Research Hospital, Izmir, Turkey

**Keywords:** Breast cancer, Discordance, Hormone receptor, Her-2, Prognosis, Diseases, Oncology

## Abstract

Biomarkers such as hormone receptors (HR) and human epidermal growth factor receptor2 (HER2) may change after neoadjuvant chemotherapy (NAC) in breast cancer patients. The aim of this study was to investigate the rates of receptor change after NAC and to evaluate the prognostic impact of change. Patients with breast cancer who received NAC were included in the study. Changes in pathological findings (ER, PR, HER-2, Ki-67, grade) before and after NAC were examined. In addition, the effect of receptor exchange on prognosis was evaluated. Kaplan Meier analysis was used for survival analyses. Study was approved by Ethics Board of Tepecik Training and Research Hospital (Decision number 2021/10-02). We confirm that all methods were performed in accordance with relevant named guidelines and regulations. The study included 203 female patients. When pathological findings before and after NAC were compared, significant regression was found in grade and Ki-67 values (*p* = 0.003, *p* < 0.001). ER change rate was 11.8%, PR change rate was 24.6% and HER-2 change rate was 12.5%. No significant correlation was found between ER, PR and HER-2 changes and prognosis. The pathological T stage after NAC being 1 or 2, no lymph nodes detected, and the tumor grade being 1 or 2 were independent variables related to survival (*p*: 0.002, *p*: 0.014, *p* < 0.001). In patients with breast cancer, it would be appropriate to re-evaluate the HER-2 and HR status of the surgical specimen following NAC, especially in initially negative patients. The correlation of receptor discordance with prognosis is not clear and more extensive studies are needed.

## Introduction

Breast cancer (BC) is the most common cancer in women worldwide. With more than two million new cases each year; it accounts for almost one in four cancers in women^1^. Neoadjuvant chemotherapy (NAC) is widely recognised as an important treatment option for early stage and locally advanced breast cancer patients with similar long-term clinical outcomes to adjuvant therapy^2^. In addition to reducing tumour stage, NAC offers the opportunity to test chemotherapy response in vivo. Patients who achieve a pathological complete response (pCR) through NAC have a better prognosis than those with residual disease^3,4^.

Core biopsy is commonly performed in the initial evaluation of breast cancer to confirm the diagnosis and determine the status of immuno-histochemical (IHC) markers. The biopsy identifies important factors such as hormone receptor [estrogen(ER) and progesterone (PR)] and human epidermal growth factor receptor 2 (HER-2), which provide insights into treatment choice and prognosis^5^. Therefore, core biopsy before treatment is very valuable in terms of determining the treatment plan and providing information about prognosis. In many studies, it has been found that there are differences in ER, PR and HER-2 expression between the biopsy taken before NAC and the surgical material obtained after NAC^6–8^. It is currently unknown how NAC modulates these biomarkers. Differential expression of treatment-directed biomarkers in tumour tissue before and after NAC may provide important additional information that may improve treatment management. Thus far, little is known about the prognostic value of biomarker differentiation after NAC. Some studies have attempted to correlate receptor changes with treatment response, but with conflicting results^9^. Retrospective analyses of primary and recurrent breast cancers revealed that receptor discordance is associated with prognosis^10^.

Receptor conversions are also common in patients not receiving NAC, but are more frequent in patients receiving NAC^11^. However, the impact of these conversions on subsequent treatment options and how they affect the prognosis of patients is still unclear.

The aim of this study was to evaluate the HR and HER-2 discordance status in patients with residual tumours after NAC and to investigate its effect on prognosis.

## Material and method

The study population consisted of 476 breast cancer patients admitted to Dokuz Eylül University Hospital Medical Oncology and Izmir Tepecik Training and Research Hospital Medical Oncology outpatient clinics between 2007 and 2022. We excluded 139 patients with pCR after NAC and 136 patients with residual disease but inadequate hormone receptors and HER-2 status in the post-operative pathology specimen. A total of 203 patients met the inclusion criteria and were included in the final analysis. Demographic, clinical, pathological and radiological characteristics of the patients were recorded. The pathological characteristics of ER, PR and HER-2 were examined for changes in core biopsy and surgical specimens. Each receptor was evaluated separately for each of the three receptors in terms of change status. Disease free survival (DFS) and overall survival (OS) data of patients with altered receptor status were analysed compared to patients without altered receptor status.

Patients with distant metastatic disease, neoadjuvant endocrine therapy and male breast cancer were excluded. Breast cancer samples with 1–100% of tumor nuclei positive should be interpreted as ER positive. However, the Expert Panel acknowledges that there are limited data on endocrine therapy benefit for cancers with 1% to 10% of cells staining ER positive. Samples with these results should be reported using a new reporting category, ER Low Positive, with a recommended comment. A sample is considered ER negative if < 1% or 0% of tumor cell nuclei are immune reactive. Additional strategies recommended to promote optimal performance, interpretation, and reporting of cases with an initial low to no ER staining result include establishing a laboratory-specific standard operating procedure describing additional steps used by the laboratory to confirm/adjudicate results. The status of controls should be reported for cases with 0% to 10% staining. Similar principles apply to PR testing, which is used primarily for prognostic purposes in the setting of an ER positive cancer^12^. The percentage of tumor cells expressing the ER or PR receptor in the nucleus of the cells was categorized according to receptor staining strength (weak, moderate, strong; negative to 3+). HER-2 staining level was scored 0, 1+ , 2+ , and 3+ . 0: defined as no staining or weak cytoplasmic and/or incomplete membranous staining in less than 10% of invasive tumour cells; +1: Defined as faint or barely visible, incomplete membranous staining in more than 10% invasive tumour cells; +2: Defined as weak to moderate complete membranous staining (considered equivalent) in more than 10% invasive tumour cells; +3: defined as intense complete membranous staining in more than 10% of invasive tumour cells. While score 0 and +1 cases were considered negative, HER-2 silver in situ hybridization (SISH) values were obtained from the hospital registration system to confirm HER-2 gene amplification in score +2 cases. Score +3 cases were considered HER-2 positive^13^. Patients received NAC according to their clinical and pathological characteristics in accordance with the recommendations of the treating physicians. A standard protocol was not applied. Mostly anthracycline + taxane based protocols were applied. HER-2 positive patients were given anti- HER-2 treatment (trastuzumab or trastuzumab + pertuzumab) along with NAC. HR positive patients received adjuvant endocrine therapy according to their menopausal status. In HER-2 positive patients, trastuzumab was completed for 1 year after surgery. Complete pathological response was defined as the absence of residual tumor cells in the breast and axilla after completion of NAC. Demographic data and menopausal status of the patients were recorded.

SPSS 25.0 (IBM Corporation, Armonk, New York, United States) package programe was used to analyse the variables. The conformity of univariate data to normal distribution was evaluated by Shapiro–Wilk test, conformity of multivariate data to normal distribution was evaluated by Mardia; (DornikandHansen omnibus) test and homogeneity of variance was evaluated by Levene test. According to the normal distribution and homogeneity of variance of the data, appropriate parametric and nonparametric analyses were applied. Quantitative variables were expressed as mean ± SD (standard deviation) and median range (Maximum-Minimum) and categorical variables were expressed as n (%).The Kaplan–Meier method was used for survival analysis and a log-rank test was performed to compare survival in different groups. Overall survival (OS) was defined as the duration from the date of diagnosis to death or last follow-up, with no restriction on the cause of death. Univariate and multivariate analysis were performed to determine the effect of independent risk factors on prognosis using the Cox-regression method. Variables were analysed at 95% confidence level and p value less than 0.05 was considered significant.

### Ethical approval

The study was approved by Ethics Board of Tepecik Training and Research Hospital (Decision number 2021/10-02).

### Patient consent

Since the study was a retrospective archive search, informed consent was not obtained from the patients.

## Results

The study included 203 female patients. The mean age was 51.5 ± 11.4 years (range: 26–78). 82.8% of the patients were over 40 years of age and 54.7% were postmenopausal. ECOG performance score was 0 in 91.5%, 1 in 8%, and 2 in 0.5%.According to the initial core biopsy results, the clinical subtype was Luminal in 155 patients (76.4%), HER-2 enriched in 16 patients (7.9%) and triple negative in 32 patients (15.8%). The median follow-up period was 31.9 months. At the end of the follow-up period, recurrence or metastasis developed in 40 patients (19.7%) and mortality occurred in 24 patients (11.8%). Clinical and pathological characteristics of the patients are shown in Table 1.

**Table 1:** Clinical and pathological characteristics of the patients.

Parameter	Sub-group	n	%
Age	≤ 40	35	17.2
	> 40	168	82.8
Menopausal state	Premenopausal	92	45.3
	Postmenopausal	111	54.7
cT	T1	28	13.8
	T2	109	53.7
	T3	39	19.2
	T4	27	13.3
cN	N0	20	9.9
	N1	109	53.7
	N2	64	31.5
	N3	10	4.9
ypT	T1	138	69.3
	T2	46	23.1
	T3	11	5.5
	T4	4	2
ypN	N0	71	35.7
	N1	72	36.2
	N2	48	24.1
	N3	8	4
Pre Ki-67	High (≥ 15%)	191	94.1
	Low (< 15%)	12	5.9
Post Ki-67	High (≥ 15%)	103	57.5
	Low (< 15%)	76	42.5
Focality	Unilocal	136	67.3
	Multifocal	32	15.8
	Multicentric	11	5.4
	Multifocal + multi-centric	23	11.4
Histology	IBC-NST	165	81.3
	ILC	13	6.4
	Other	25	12.3
Grade	1	4	2
	2	120	60.3
	3	75	37.7
Surgery type	BCS	81	39.9
	MRM	78	38.4
	Simple Mastectomy	37	18.2
	Bilateral MRM	3	1.5
	Bilateral symple mastectomy	4	2

*T* tumour, *N* node, *ILC* invasive lobular carcinoma, *IBC-NST* invasive breast carcinoma no special type, *BCS* breast conserving surgery, *MRM* modified radical mastectomy.

Changes in receptor and pathological features evaluated in core biopsy and surgical specimens are shown in Table 2. Tumour grade and Ki-67 proliferation index were statistically significantly lower in surgical specimens after NAC.

**Table 2:** Pre-op and post-op changes in pathological features.

Features	Pre		Post		p*
	n	%	n	%	
Grade					
1	4	2.0	118	58.1	**0.003**
2	120	60.3	19	9.4	
3	75	37.7	34	16.7	
ER status					
Negative	49	24.1	32	15.8	0.600
Pozitive(≥ 1)	154	75.9	171	84.2	
1+	10	4.9	118	58.1	
2+	19	9.4	19	9.4	
3+	125	61.6	34	16.7	
PR status					
Negative	77	37.9	32	15.8	0.091
Pozitive(≥ 1)	126	62.1	171	84.2	
1+	14	6.9	118	58.1	
2+	27	13.3	19	9.4	
3+	85	41.9	34	16.7	
HER-2 status					
Negative	118	58,1	32	15,8	0.104
1+	19	9.4	118	58.1	
2+	34	16.7	19	9.4	
3+	32	15.8	34	16.7	
Ki-67(mean ± SD)	39.6 ± 21.4	(3–90)	24.7 ± 22.7	(1–95)	** < 0.001**

*ER* oestrogen, *PR* progesterone, *HER-2* human epidermal growth factor 2; The percentage of tumor cells expressing the ER or PR receptor in the nucleus of the cells is recorded along with assessment of receptor staining strength (negative, weak, moderate, strong). Staining strength is scored from negative to 3 +

*McNemar–Bowker Test.

Significant values are in (bold).

Among 203 patients, 127 (62.6%) had no receptor change. In 58 patients (28.6%), only one receptor was changed. 14 patients (6.9%) had changes in 2 receptors and 4 patients (2%) had changes in all three receptors. ER status changed in a total of 24 patients (11.8%). Among these, 10 patients changed from positive to negative and 14 patients changed from negative to positive. PR status changed in 50 patients (24.6%). 29 patients changed from positive to negative and 21 patients changed from negative to positive. HER-2 status changed in 26 patients (12.5%). Among these, 19 patients changed from positive to negative and 7 patients changed from negative to positive.

**Figure 1:**
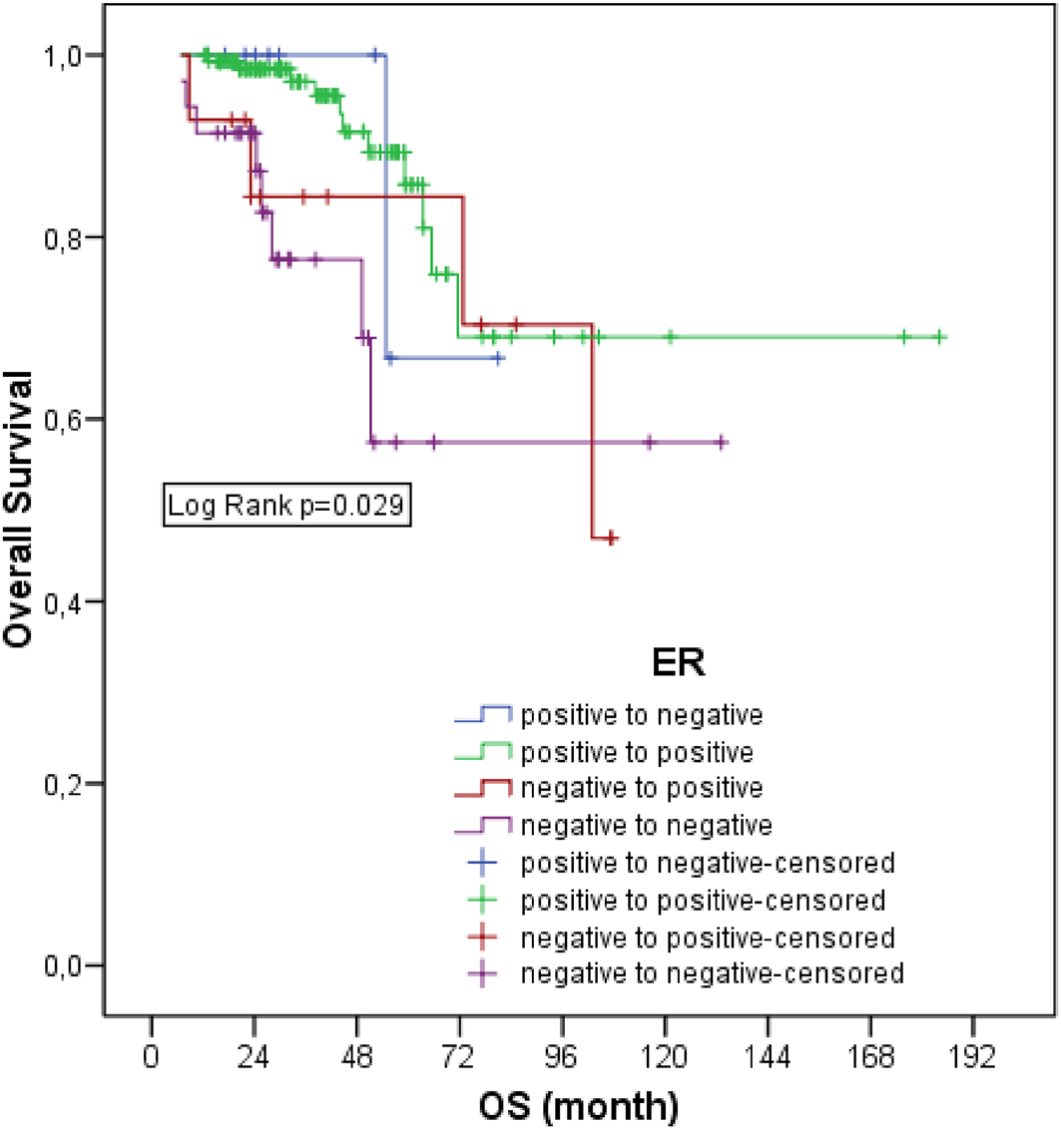
Overallsurvival of patients according to ER status.

The 5-year survival rate was 87.5% in patients whose ER status was positive and did not change, 66.7% in patients whose ER status was positive and turned negative, 84.4% in patients whose ER status was negative and turned positive, and 57.4% in patients whose ER status was negative and did not change (*p* = 0.029). Survival rates of the ER negative group who remained negative after NAC were statistically significantly lower than those who remained positive (*p* = 0.002). No significant difference was found in patients with ER change compared to those without (Fig. 1).

Three-year DFS was 75.6% in those whose ER status was positive and did not change, 56.8% in those whose ER status was negative and did not change, 66.7% in those whose ER status was positive and turned negative, and 64.9% in those whose ER status was negative and turned positive, and there was no statistically significant difference (*p* = 0.387).

**Figure 2:**
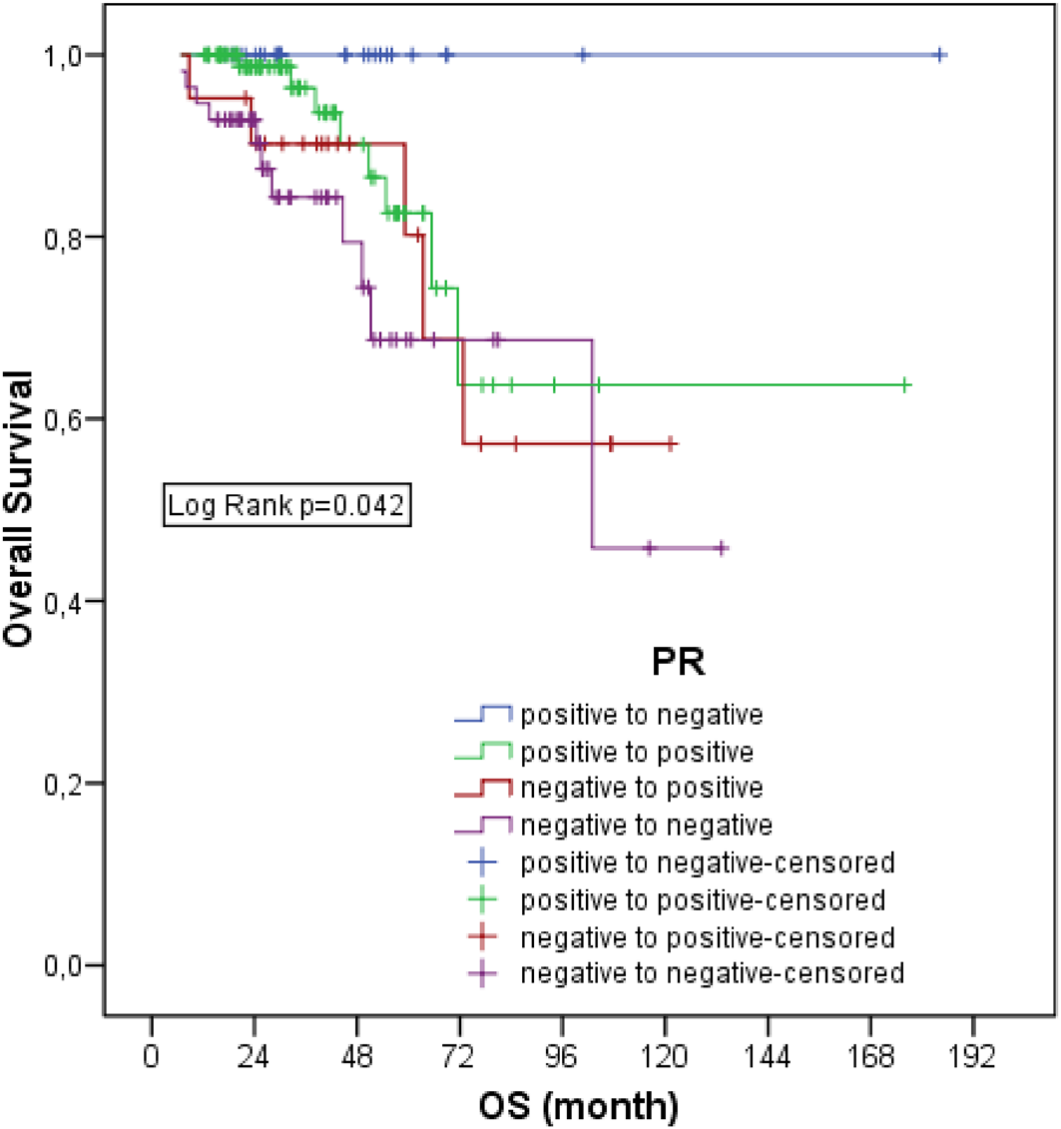
Overall survival of patients according to PR status.

According to the PR status, the 5-year survival rate was 82.6% in those who were positive and did not change, 68% in those who were negative and did not change, 100% in those who changed from positive to negative, and 80.2% in those who changed from negative to positive (*p* = 0.042). Survival rates of the groups with PR change from negative to negative and negative to positive were statistically significantly lower than those with positive to negative (*p* = 0.011 *p* = 0.049) (Fig. 2).

Five-year DFS rates were 51.8% in PR positive patients who did not change, 47.4% in negative patients who did not change, 73.8% in positive patients who turned negative, and 51.8% in negative patients who turned positive and there was no statistical significance (*p* = 0.696).

**Figure 3:**
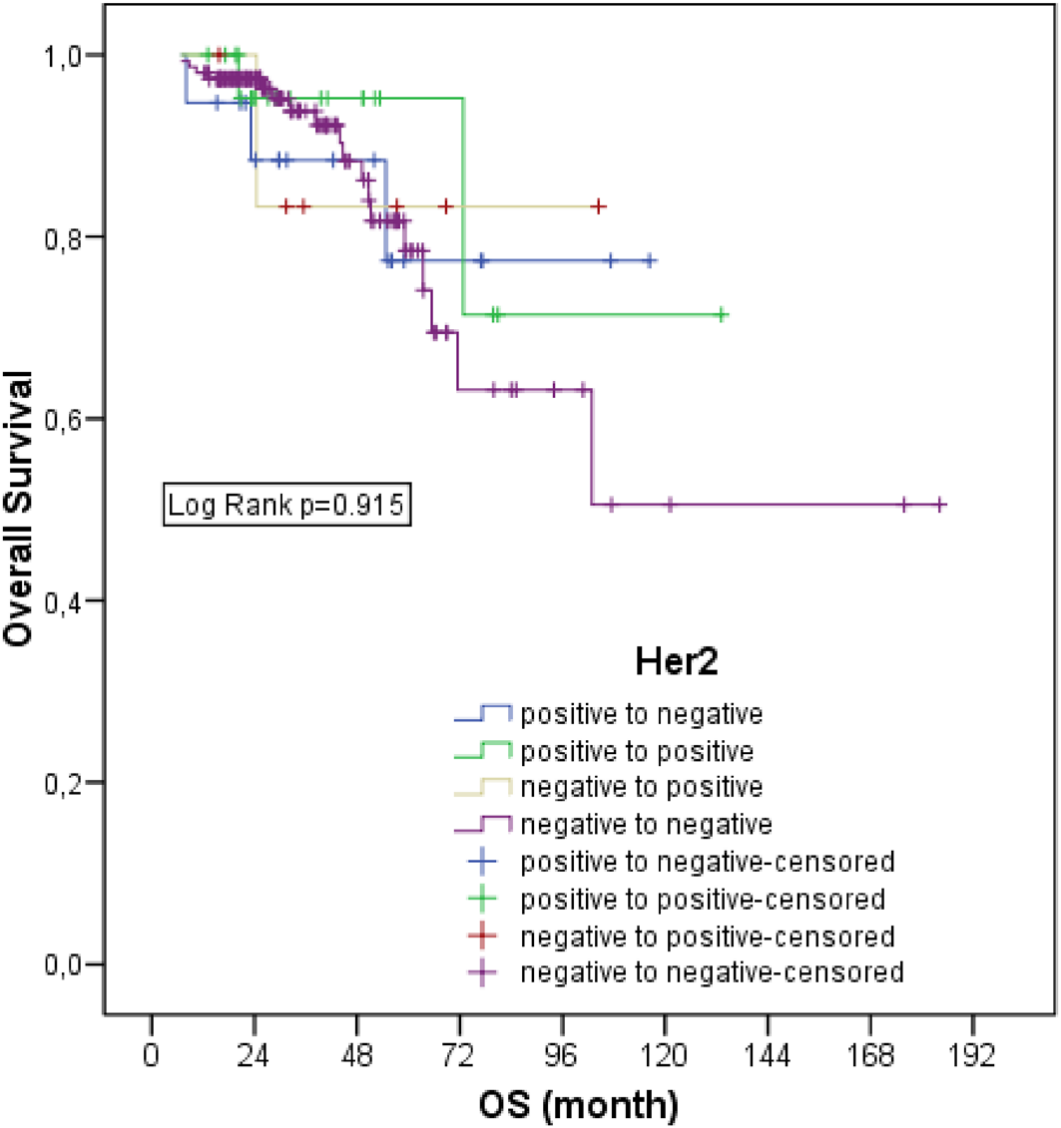
Overall survival of patients according to HER-2status.

Five-year survival was 95.2% in HER-2 positive and unchanged, 77.4% in HER-2 positive and changed to negative, 83% in HER-2 negative and changed to positive, and 78.5% in HER-2 negative and unchanged. There was no statistically significant difference in survival rates in HER-2 change groups (*p* = 0.915) (Fig. 3).

Five-year DFS was 40.1% in HER-2 positive patients who did not change, 78.6% in those who turned positive to negative, 62.4% in those who turned negative to positive, and 54.4% in those who turned negative to positive and did not change, and was not statistically significant (*p* = 0.816). Intergroup survival rate analyzes according to ER, PR and HER-2 status are presented in Table 3.

**Table 3:** Intergroup survival rate analyzes according to ER, PR and HER-2 status.

	Positive to negative	Positive to positive	Negative to positive
	*p*	*p*	*p*
ER status			
Positive to positive	0.810		
Negative to positive	0.756	0.399	
Negative to negative	0.254	**0.002**	0.694
PR status			
Positive to positive	0.089		
Negative to positive	**0.049**	0.591	
Negative to negative	**0.011**	0.069	0.603
HER-2 status			
Positive to positive	0.641		
Negative to positive	0.874	0.690	
Negative to negative	0.837	0.452	0.777

*ER* oestrogen, *PR* progesterone, *HER*-2 human epidermal growth factor 2.

Significant values are in (bold).

## Discussion

Neoadjuvant chemotherapy is an important treatment modality that offers advantages such as making an inoperable tumour operable, eliminating micrometastatic disease, observing the in vivo response of the tumour to systemic therapies and is widely used in breast cancer treatment^14^. In addition, in recent years, the treatment of residual disease after NAC in HER-2 positive and triple negative breast cancer has been shown to contribute to survival, and NAC applications have become more preferred and applied in earlier stages^15,16^.

The NAC plan is based on the HR and HER-2 status determined by the initial core biopsy. Re-evaluation of receptor status from the postoperative material is recommended especially for patients with negative receptor status at baseline^17^. Core biopsy material represents a pathologically limited evaluation area. Surgical material provides the opportunity to evaluate a more comprehensive and heterogeneous tumour area. There are various explanations for discordance between core biopsy and resection specimen biomarker profiles in breast cancer, including tumor heterogeneity and pre‐analytic variation. It is also thought that ER/HER-2 activity can be lost during the time between tumor acquisition and fixation^18^. Seferina et al. compared the breast core needle biopsy and the resection specimen with respect to ER, PR and HER-2 status. They found that the assessment of ER by IHC in core needle biopsy was false negative in 26.5% and false positive in 6.8% of patients. For the PR status the false negative and false positive results of core needle biopsy were 29.6% and 10.3%, respectively. The results of the HER-2 status, as determined by IHC and SISH, were false negative in 5.4% and false positive in 50.0%. They drew attention to this receptor discordance in patients without neoadjuvant therapy^19^. For this reason, the receptor status, which is initially negative, may be found positive in the surgical material and this requires a change in adjuvant treatment. This may affect the prognosis of the patient. It is also possible for an initially positive receptor to be negative in the surgical material. Theoretically, this change can be attributed to the elimination of tumour clones with the positive receptor as a result of treatment. In such cases, adjuvant treatment is performed according to the positive result and no treatment change is made. The effect of these approaches on prognosis is still unclear.

The mechanism of conversion of HR and HER-2 after NAC is not clearly known. Several different clones with different phenotypes may cause the presence of intra-tumour heterogeneity. Within the same tumour, some clones may be HR(+) and some may be HR (−). Likewise, HER-2 (+) and (−) cells are not evenly distributed within the tumour. Sensitivity to chemotherapy is different between different clones. HR (−) tumour cells are more sensitive to chemotherapy than HR (+) tumours. Vemuru et al. found that HR+ tumors were more likely than HR− tumors to have a receptor conversion (37% vs 11%; *p* < 0.01). Furthermore, patients with stage 0-II disease after NAC were more likely to have receptor conversions than those with stage III-IV disease (35% vs 13%; *p* = 0.02)^20^. Previous studies on the change in HR and HER-2 status after NAC have presented conflicting results. Chen et al. and Van de ven et al. showed that there was a change in tumour receptor expression after NAC^21,22^. Arens et al. argued in their study that patients did not show receptor discordance after NAC^23^. In our study, we found varying rates of HR and HER-2 discordance. In a review, it was concluded that the rates of ER, PR and HER-2 discordance were 5.9–51.7%, 2.3–35% and 2.5–17%, respectively^22^. A large-scale retrospective study reported that approximately 21.4% of HER-2 positive breast tumour patients had HER-2 conversion to negative^24^. In our study, this discordance was highest in the progesterone receptor with a rate of 23.1%. Gupta et al. reported that ER discordance was found to be 8.69% (P value 0.76), PR discordance was 17.39% and PR discordance was found to be higher than ER discordance^25^. In general, our receptor discordance rates were compatible with the literature.

In our study, when survival was evaluated according to ER receptor status, ER positive patients who remained unchanged after NAC had the best 5-year survival (87.5%). Patients who changed from negative to positive ranked second, and patients who changed from positive to negative ranked third. Patients who were ER negative and did not change after NAC had the worst 5-year survival (57.4%). Statistically significant differences were found between these groups. However, when we looked at the pairwise comparisons between the groups, we observed that the significance was due to the patients who were positive and remained positive and the patients who were negative and remained negative. In fact, the difference was due to patients who did not show discordance and it is an expected result that the survival of ER positive patients is better than ER negative patients.Therefore, ER change did not show any difference in OS in our study. In Tacca et al. study, the survival of patients with HR change was compared with that of patients without change, and in this comparison, those who were negative and remained negative were taken as the unchanged group. In this study, the overall survival of both those who were HR positive and turned negative and those who were negative and turned positive were significantly higher than those who were negative and remained negative^26^. In fact, although statistical significance could not be shown in our study, the groups had a similar ranking in terms of survival. This may be related with the smaller number of our patients. In the study by Ding et al., it was shown that conversion from positive to negative in HR was associated with poor survival^27^.

In our study, we found that the survival of patients who changed from PR positive to negative was better than the PR negative and unchanged group and the PR negative to positive group. This was not aresult we expected. Although it was statistically significant, we thought that this result might be coincidental. In addition, we analysed survival separately for each receptor change in our study. However, 6.9% of the patients had changes in 2 receptors and 2% had changes in all three receptors. Multiple receptor changes may also have affected the results. In addition, we know that ER is a more dominant determinant for hormone receptor positivity. Therefore, it is not rational that ER change is not significant and PR change is significant.

In our study, we also found that HER-2 discordance did not affect survival. Tural et al. reported that loss of HER-2 receptor is associated with poor outcome and is an independent risk factor for DFS^28^. Wang et al. associated patients whose HER-2 status changed from positive to negative with a 2.64 times (95% CI 1.10–6.31) increased risk of recurrence compared with patients who were HER-2 positive and remained positive^29^. On the contrary, we found that HER-2 conversion alone had no effect on prognosis in our study. In Wang's study, all patients were initially positive and represented a more homogenous group. In our study, all patients who were both positive and negative at baseline were included. Patients who were HER-2 positive in either core biopsy or surgical material received anti HER-2 therapy. We think that survival may not have differed between the groups for this reason. In the past, HER-2 positivity was associated with poor survival, but nowadays, with the development and increasing use of anti HER-2 therapies, survival has become similar to the luminal B subtype.

In our study, ER, PR and HER-2 change status were not found to be associated with survival in univariate analysis. Therefore, it was not included in multivariate analysis. Among the parameters that were associated with survival in univariate analysis and evaluated in multivariate analysis, we found that the decrease in pathological T and N stages after NAC and the decrease in tumor grade after NAC were independent variables associated with good survival. It is a predicted condition for breast cancer that the patient with a good pathological response will have a good survival. However, in our study, we found that the initial ER, PR status, and indirectly the clinical subtype, was the only dependent variable for survival. Univariate and multivariate analysis for overall survival presented in Table 4. These results showed us that the treatment response achieved with NAC is still the most important prognosis determinant in breast cancer patients receiving NAC. In this study, where we investigated the relationship between receptor change and prognosis, our data suggested that there is no relationship between receptor change and prognosis, and even if the receptors do not change, conditions associated with poor prognosis in breast cancer, such as ER and PR negativity, are less important than the pathological response in determining prognosis.

**Table 4:** Univariate and Multivariate Cox Regression Analysis for Survival.

Characteristics	Univariate analysis		Multivariate analysis	
	5 year OS (%)	*p* value	Adjusted HR (95%CI)	*p* value
Menopausal status				
Premenopause	82	0.933		
Postmenopause	94			
Clinical T stage				
1 and 2	97	**0.009**		
3 and 4	75			
Clinical N stage				
N0	90	0.602		
N+	82			
Core biopsy ER status				
Positive	84	**0.007**		
Negative	68			
Core biopsy PR status				
Positive	87	**0.017**		
Negative	71			
Core biopsy Her-2 status				
Positive	84	0.605		
Negative	78			
Core biopsy tumor grade				
1–2	92	0.215		
3	84			
Core biopsy Ki-67 status				
< 15	100	0.184		
≥ 15	79			
ypT status				
0–1	89	** < 0.001**	4.1 (1.6–10.1)	**0.002**
2–4	63			
ypN status				
N0	94	**0.013**	6.4 (1.4–28.5)	**0.014**
N+				
Surgical specimen tm grade				
1–2	84	** < 0.001**	5.4 (2.1–13.6)	** < 0.001**
3	72			
Surgical specimen Kİ-67				
< 15	92	0.074		
≥ 15	68			
Surgical specimen ER status				
Positive	86	**0.017**		
Negative	58			
Surgical specimen PR status				
Positive	81	0.604		
Negative	79			
Surgical specimen Her-2 status				
Positive	91	0.550		
Negative	78			
ER discordans				
No	80	0.659		
Yes	80			
PR discordans				
No	77	0.224		
Yes	88			
HER-2 discordans				
No	80	0.859		
Yes	78			

*Tm* tumour, *N* node, *ER* estrogen, *PR* progesterone, *HER-2* human epidermal growth factor 2, *yp* pathological staging after neoadjuvant therapy.

Significant values are in (bold).

The most important limitation of our study was that it was performed in a very heterogeneous group. All patients who received NAC were included and the evaluation was performed individually at each receptor status. For example, a patient who turns from ER negative to positive may be triple negative or HER-2 enriched type at the beginning. There will be many confounding factors when comparing the effect of receptor changes in these two groups on survival. Similar evaluations in more homogenous groups such as luminal type or triple negative type with a larger number of patients may provide more accurate results. Another limitation of our study was that it was retrospective and the number of patients was small.

Our study also showed that receptor discordance rates were not low after NAC and underlined the need to re-evaluate receptors in surgical specimens. Awareness on this issue is important for adjuvant treatment optimization. In addition, our study shed light on more comprehensive studies on this subject.

## Conclusion

Following neoadjuvant therapy, re-detection of HER-2 and HR status of the surgical specimen and comparison with the results obtained with the initial core biopsy may be useful for optimisation of adjuvant therapy. The effect of receptor discordance on prognosis remains unclear. Larger studies in more homogeneous patient groups are needed to explore therapeutic strategy and to determine more effective prognosis.

## Data Availability

Te datasets used and/or analysed during the current study available from the corresponding author on reasonable request.
